# United States Army Veterinary Service

**Published:** 1888-01

**Authors:** 


### UNITED STATES ARMY VETERINARY SERVICE.

This will be the object of a bill to be presented to the Congress at Washington during the present session and it should receive the most heartfelt sympathy and support from all of our readers. A great deal must be asked for and every veterinarian in the country must make up his mind that what is asked for must be obtained. Every one knows some member or senator who should be seen at once so that he will give the subject consideration and understand its importance when it is presented, so that his friend the member will be ready to favor any amendments which will increase the demands of the bill, and discourage any curtailing of its provisos. Many of the medical officers of the Army as well as others of the Line favor and will aid the passage of a good act. Show each of your agricultural friends that an Army Service on a high standard means not only the saving of many times its cost to the War Department, but means the dissemination over the country of educated men who will be pioneers for the development of better local practitioners. To the Department of Agriculture, and to the cattle men of the West, it means a large force of spies who will be on the alert for the first appearance of any contagious disease or epizootic among the immense herds that surround the scattered army posts the other side of the Mississippi. H.

---

AMERICAN VETERINARY REVIEW PRIZE.—The editorial staff of the *American Review* offer a prize of one hundred dollars for the best original paper on any subject pertaining to veterinary science. The prize will be awarded upon the verdict of a committee of gentlemen selected by the editors of the *Review.*

It is needless to remark on the advantages to be derived from this plan of awakening effort in the direction of original work. Competitors for this premium must forward their contributions for publication to the office of the *Review*, 141 West 54th St., New York City, before the first of April, 1888, each paper being distinguished by a special motto and accompanied by a sealed envelope, enclosing the name and address of the author, and endorsed externally with the distinguishing motto for identification. C.

---

PARASITES IN THE WILD ASS.—V. S. John H. Steel, A. D. V. Supt. Bombay Vet. Coll., publishes in the Journal of the Natural History Society of that city a list of parasites found in the body of a wild ass. This examination was made by Dr. Steel as he said, he was opposed to the popular view that these parasites do not occur in wild animals to such a degree as in domesticated. The result was as follows :

*Stomach.*—Cysts and spiroptera either from these cysts or from the species which inhabit the stomach cavity. Bots, *Ascaris megalocephala.*

*Small Intestines.*—*Ascaris megalocephala* in enormous numbers.

*Cœcum and commencement of Colon.*—Cysts with small white worms. *Strongylus armatus.*

*Rectum.*—*Oxyurides.*

*Anterior mesenteric Artery.*—One immature *Strongylus armatus.*

The parasites found being of six different species. The following were some of the points deduced: That the parasites above mentioned must be obtained by animals out on natural pasturage. This is likely to be a useful hint as regards their prevention in domestic equines. That parasitic invasion is not by any means an inflection in animals following solely in the train of domestication and that parasites which infest the wild ass are of the same species as those found in the domesticated horse. C.

---

OBSERVATIONS ON THE PYTHON.—Mr. H. M. Phipson, Hon. Secretary of the Bombay Natural History Society, has made some interesting observations in the feeding and temperature of the Indian Rock Snake *Python molurus*, which is published in the journal of the above society. During the twelve months between May 27, 1886, and May 20, 1887, the snake ate twenty-three rats, three hens, three crows and one hawk. In the hot months the period of digestion averaged about eight days, whereas in the cold weather it became much slower, in one instance being thirty-eight days. During the cold weather for a period of one hundred and thirteen days, the snake refused food and remained in a very sluggish, and sleepy condition. During this period of the hybernation the temperature of the reptile fell from 82° normal to 73°, a fall of nine degrees. The snake shed its skin four times in the course of the year ; three times in hot weather at an interval of two months, and once after it had recovered from its hybernation. C.

---

PLEURO-PNEUMONIA.—At a recent meeting of the Veterinary Science Association a paper on the above subject, by Dr. J. W. Gadsden, M. R. C. V. S., was omitted for want of time.

In this paper Dr. Gadsden shows how Pleuro-Pneumonia is contagious and incurable, and states as the result of his experience and observation, that the contagion can only be communicated by contact with the living diseased animal, and in this respect it differs from all other contagious diseases of cattle. Many foreign Veterinarians are in accord with him in this opinion.

---

NOTES.—Experiments have recently been made in London to see what effect the inoculation of muscle juice from cases of Black leg, dried in a current of air at various temperatures, would have upon animals.

Muscle juice dried at a temperature of 32°c. had no effect upon either guinea pigs or cattle. Intravenous inoculation of fresh muscle juice from cattle which had just died of the disease did not kill the animals so inoculated and they afterward stood a subcutaneous inoculation of fresh muscle juice, while it proved fatal to those animals which had not previously been intravenously inoculated.

---

Professor Schütz, of Berlin, has recently been in London to prove the identity of the swine plague in England and Germany. He considers the disease to be the same in both countries.

---

The loss to the corps of instruction of the Veterinary Profession, so recently made by the retirement of M. Goubaux, the eminent Anatomist and respected Director of the Alfort School in France, is followed by one of a more active teacher, whose separation from his laboratory will be learned with regret, by the experimental world as well as by the veterinarian. M. Nocard the present Director of the Alfort School in announcing the retirement of Professor Colin the Physiologist, and his appointment as Honorary Professor of the Veterinary Schools of France, concluded with the following words: "No one merited better "this title than the eminent savant who, during long "years has been the honor of the Veterinary Profession and "the pride of the School of Alfort. Our respectful admir-"ation will accompany him in his retirement, and we will "keep as a precious example, the recollection of his forty "years of inveterate labors and imperishable work."

---

We take pleasure in informing our readers that Dr. William Herbet Lowe, D.V.S., of Paterson, Secretary of the New Jersey State Veterinary Society, and one of the State Veterinary Inspectors, has been appointed Superintendent of the United States Cattle Quarantine Station for the port of New York.

---

The *Medical Times*, a bi-weekly Journal of Medicine and

Surgery, formerly published by the J. B. Lippincott Co., is now edited and published by Dr. Frank Woodbury and Dr. William F. Waugh. Dr. Woodbury delivered the address in Medicine before the State Medical Society of Pennsylvania, at its last annual session. Dr. Waugh was formerly on the editorial staff of *The Medical World.* Both gentlemen hold professorships in the Medico-Chirurgical College of Philadelphia. The *Times* shows many improvements in its recent issues and is now one of the leading medical journals of this country.

---

The *Archives of Pediatrics*, published by J. B. Lippincott Company of Philadelphia, and edited by William Perry Watson, A.M., M.D., in the issue of the January number, 1888, will begin a series of articles on the Therapeutics of Infancy and Childhood, by A. Jacobi, M.D., Clinical Professor of Diseases of Children in the College of Physicians and Surgeons, President of the New York Academy of Medicine.

